# The Sensitivity and Specificity of White Blood Cells and Nitrite in Dipstick Urinalysis in Association With Urine Culture in Detecting Infection in Adults From October 2016 to October 2019 at King Abdulaziz Medical City

**DOI:** 10.7759/cureus.15436

**Published:** 2021-06-04

**Authors:** Abdulrahman T Mohanna, Khalid M Alshamrani, Majd A SaemAldahar, Abdulbari O Kidwai, Abdulrahman H Kaneetah, Mohammed A Khan, Nadia Mazraani

**Affiliations:** 1 College of Medicine, King Saud Bin Abdulaziz University for Health Sciences, Jeddah, SAU; 2 College of Medicine, King Abdullah International Medical Research Center, Jeddah, SAU; 3 Medical Education, King Saud Bin Abdulaziz University for Health Sciences, Jeddah, SAU; 4 Medical Education, King Abdullah International Medical Research Center, Jeddah, SAU; 5 Family and Community Medicine Department, Ministry of the National Guard - Health Affairs, Jeddah, SAU

**Keywords:** urinalysis, urine culture, leucocyte, nitrite, urinary tract infection, saudi arabia, sensitivity, specificity

## Abstract

Background

Urinary tract infection (UTI) is one of the most common clinical presentations that exhaust the patients and confuse physicians. Some of the risk factors that contribute to UTIs are age, female gender, and diabetes. Urinalysis is used to detect abnormalities in the urine, such as the presence of leukocytes, blood, and nitrite. However, urinalysis accuracy depends on the patient and the analyzer. On the other hand, urine culture is considered gold standard for diagnosing UTI. For that, the aim of this study is to determine the sensitivity of white blood cells (WBC) and nitrite in dipstick urinalysis in detecting UTI.

Methods

A cross-sectional study was conducted at King Abdulaziz Medical City on adult patients aged 19-65 years who underwent dipstick urinalysis and culture at the same visit from October 2016 to October 2019. The data were collected from the medical records from all the departments by using a data collection sheet through Best Care system. The sample was selected conveniently, and it was determined to be 359 patients with a confidence interval of 95%. Data were analyzed using IBM SPSS version 20 (IBM Corp., Armonk, NY, USA). Chi-square test was used to analyze the association between the outcome and the results of the dipstick urinalysis and urine culture. P-value lower than 0.05 was considered significant.

Results

Three hundred and fifty-nine patients were included into the study with a majority of females (81.1%) with a mean age of 47.5 years. Two hundred and fifty-two patients were culture positive, WBC sensitivity and specificity were 62.7% and 100%, and nitrite sensitivity and specificity were 20.6% and 93.5%, respectively. Ninety-nine diabetic patients were culture positive; for diabetic patients, WBC sensitivity and specificity were 65.7% and 100% and nitrite sensitivity and specificity were 18.2% and 97.6%, respectively, while for non-diabetic patients, WBC sensitivity and specificity were 60.85% and 100% and nitrite sensitivity and specificity were 22.2% and 90.8%, respectively.

Conclusion

Our study showed that results of WBC are more sensitive and specific than those of nitrite in comparison to the gold standard (urine culture). Diabetics and non-diabetics have slightly different results. According to our results it is difficult to depend on the dipstick urinalysis without culture. More studies are recommended in this field.

## Introduction

Urinary tract infection (UTI) is one of the most common outpatient infections, and it is the second most commonly diagnosed infection in the acute hospital setting [1,2]. Clinical manifestations and laboratory investigations are the hands that aid physicians in diagnosing UTIs [1,3]. The symptoms of UTIs can give the patients a bitter experience that affect their quality of life and bring them to clinic or emergency department [1,3]. Dysuria, urgency, and frequency are the most common symptoms of UTIs [1,3]. Hematuria, nocturia, malodorous urine, cloudy urine, fever, and flank pain are other symptoms of UTIs [1,3]. UTIs could be classified into two categories: community-associated urinary tract infections (CAUTIs) and hospital-acquired urinary tract infections (HAUTIs) [4]. The predominant causative pathogen of UTIs is *Escherichia coli* bacteria [4]. Other pathogens include *Proteus* species, *Staphylococcus saprophyticus*, and *Klebsiella* species [4]. A study by Alanazi et al. (2016) stated that the global prevalence of CAUTIs is 0.7%, and HAUTIs frequency among health-care-associated infections is 12.9% in the United States of America, 19.6% in Europe, and 24% in developing countries [5]. The main risk factors that contribute to UTIs are age, female gender, history of UTI, and diabetes [5,6]. UTIs are associated with different complications, such as pyelonephritis, urosepsis, and hospitalization [1,2]. Different diagnostic tests are used for UTIs, but the two main tests are dipstick urinalysis and urine culture [7].

 Urinalysis is an inexpensive and convenient way to detect abnormalities in the urine [1,8]. There are multiple aspects of the urine sample to be tested, such as physical appearance of the sample, chemical composition, and microscopic examination [1,8]. Each of these aspects has its findings. The physical appearance can be tested for color, odor, and turbidity [1,8]. The chemical composition of the urine sample can be tested by the dipstick, and it checks for leukocytes, blood, nitrite, glucose, and urine pH [1,8]. The microscopic examination of the urine sample is done to check for cells including leukocytes, erythrocytes, and epithelial cells [1,8]. Microorganisms can be seen under microscope with or without the use of gram stain [1,8]. However, the urinalysis is subject to preanalytical issues like how the sample is collected, the container used, stored, and transported [9]. These issues can be corrected by patient education on how to collect the sample [9]. Clean mid-stream catch in the morning is the best way, and choosing a sterile container can give more accurate results [9]. On the other hand, urine culture is considered the traditional gold standard for diagnosing UTIs as it allows an identification of the causative agent [9]. Another important information that can be provided by the urine culture is the sensitivity of the microorganism to antibiotics [3].

 According to a study that was published in 2017, 61% of patients who had positive dipstick urinalysis had negative urine culture [1]. It reports low sensitivity and higher specificity for dipstick urinalysis [1]. After reviewing the data acquired from the literature, no local studies seem to be there discussing the sensitivity and specificity of white blood cells (WBC) and nitrite in dipstick urinalysis in comparison to urine culture in general or diabetic populations. Furthermore, the findings of this study could aid in preserving the resources and time of the healthcare system and patients, and also could lead to less antibiotic prescription. Therefore, the aim of this study was to determine the sensitivity of WBC and nitrite in dipstick urinalysis in association with urine culture in detecting infection in adults from October 2016 to October 2019 at King Abdulaziz Medical City.

## Materials and methods

**Figure 1:**
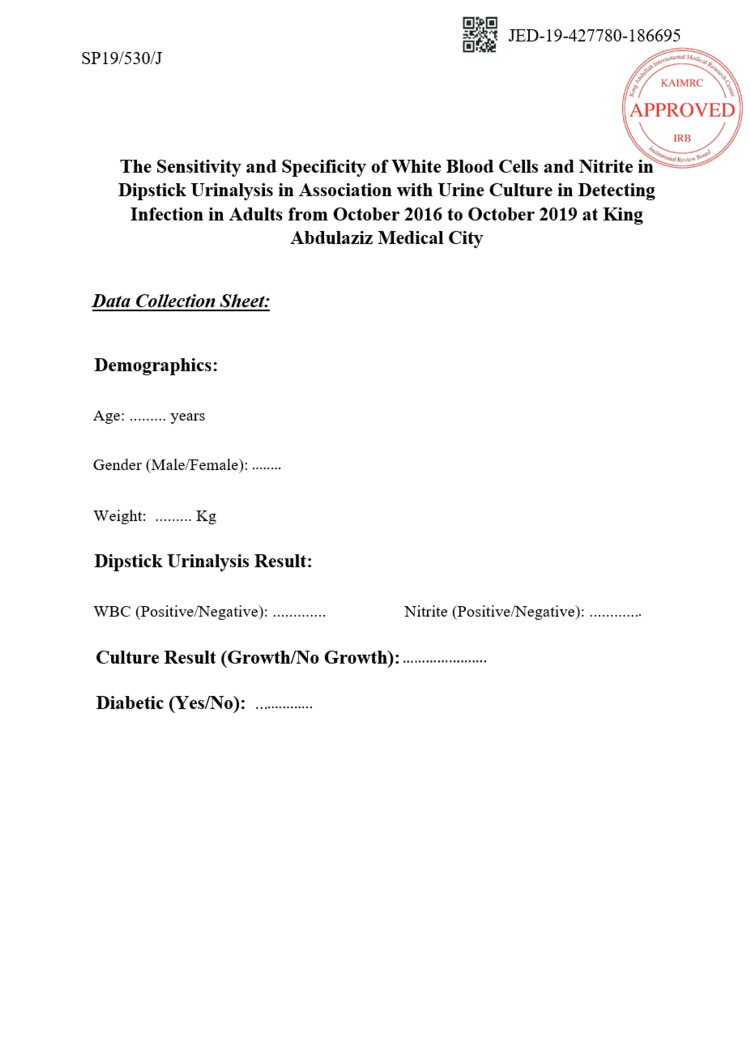
Approved Data Collection Sheet Kg: kilogram, Leu/Ul: leucocytes per microliter.

This cross-sectional study was conducted at King Abdulaziz Medical City on adult patients from October 2016 to October 2019, and the data were gathered from medical records department of King Abdulaziz Medical City. The inclusion criterion was adult patients (19-65 years) who had undergone dipstick urinalysis and culture within same visit. The data were collected using a data collection sheet ( see Figure 1 in the appendices) in which the results of the dipstick urinalysis (WBC and nitrite) and the result of urine culture (positive or negative) were collected. Other demographic variables, such as age, gender, and weight, were also collected. All data were collected through Best Care system, and were analyzed using IBM SPSS version 20 (IBM Corp., Armonk, NY, USA). Categorical data were presented as frequency and percentage. Continuous data were depicted in the form of mean and standard deviation. Chi-square test was used to analyze the association between the outcome (detecting infection) and the results of the dipstick urinalysis and urine culture. P-value lesser than 0.05 was considered as significant.

## Results

The current study involved 359 patients of the age 19-65 years. Most of them were females (n = 291; 81.1%) with a mean age of 47.5 years. Rest of the demographics are shown in Table 1. The number of positive cultures was n = 252 (70.2%), WBC sensitivity and specificity were 62.7% and 100% (p-value <0.001), respectively, and nitrite sensitivity and specificity were 20.6% and 93.5% (p-value <0.001), respectively, as presented in Table 2. For diabetic patients, the number of positive cultures was n = 99 (70.2%), WBC sensitivity and specificity were 65.7% and 100% (p-value < 0.001), respectively, and nitrite sensitivity and specificity were 18.2% and 97.6% (p-value = 0.012), respectively, as demonstrated in Table 3. For non-diabetic patients, WBC sensitivity and specificity were 60.85% and 100% (p-value < 0.001), respectively, and nitrite sensitivity and specificity were 22.2% and 90.8% (p-value = 0.023), respectively, as shown in Table 4.

**Table 1:** Demographics WBC: white blood cells; SD: standard deviation; kg: kilograms.

Demographic		Number	Percentage
Gender	Male	68	18.9
	Female	291	81.1
Diabetic	Yes	141	39.3
	No	218	60.7
WBC	Positive	265	73.8
	Negative	94	26.2
Nitrite	Positive	59	16.4
	Negative	300	83.6
Culture	Growth	252	70.2
	No growth	107	29.8
	Total	359	100
		Mean	SD
Age (years)		47.5	13.6
Weight (kg)		68.6	17.0

**Table 2:** WBC and Nitrite Results WBC: white blood cells.

		Culture		Total	p-Value
		Growth number (%)	No-growth number (%)		
WBC	Positive	158 (62.7)	107 (100)	265	
	Negative	94 (37.3)	0	94	<0.001
Nitrite	Positive	52 (20.6)	7 (6.5)	59	
	Negative	200 (79.4)	100 (93.5)	300	<0.001
Total		252	107	359	

**Table 3:** WBC Results for Diabetic and Non-diabetic Patients WBC: white blood cells.

			Culture		Total	95% Confidence interval		p-Value
			Growth number (%)	No-growth number (%)		Lower	Upper	
Diabetic	WBC	Positive	65 (65.7)	42 (100)	107	0.522	0.707	
		Negative	34 (34.3)	0	34			<0.001
	Total		99	42	141			
Non-diabetic	WBC	Positive	93 (60.8)	65 (100)	158	0.517	0.671	<0.001
		Negative	60 (39.2)	0	60			
	Total		153	65	218			

**Table 4:** Nitrite Results for Diabetic and Non-diabetic Patients

			Culture		Total	95% Confidence interval		p-Value
			Growth number (%)	No-growth number (%)		Lower	Upper	
Diabetic	Nitrite	Positive	18 (18.2)	1 (2.4)	19	1.17	70.66	0.012
		Negative	81 (81.8)	41 (97.6)	122			
	Total		99	42	141			
Non-diabetic	Nitrite	Positive	34 (22.2)	6 (9.2)	40	1.12	7.07	0.023
		Negative	119 (77.8)	59 (90.8)	178			
	Total		153	65	218			

## Discussion

In this study, sensitivity and specificity of WBC and nitrite in dipstick urinalysis were used to detect infection in adults by evaluating WBC and nitrite individually in the test results. WBC had better sensitivity compared to nitrite in this study. In contrast, specificity was higher for nitrite compared to WBC. One study conducted in Nigeria reported lower sensitivity and specificity for WBC in detecting infection, taking in consideration that their sample size was larger than our study. However, the same study reported similar result for nitrite sensitivity and specificity [10]. Another study conducted in Pakistan reported similar sensitivity and specificity for WBC but higher sensitivity with similar specificity for nitrite with a smaller sample size than our study [11].

 Furthermore, two studies conducted in Malaysia and Australia reported higher sensitivity but lower specificity for both WBC and nitrite [12,13]. Moreover, with a lower sample size, Bachman’s study conducted in the United States of America reported very low sensitivity but similar specificity for WBC in comparison to our results and higher sensitivity and specificity for nitrite [14]. Semeniuk’s study conducted in Canada reported higher sensitivity but lower specificity for WBC and very low sensitivity for nitrite with higher specificity [15]. Schwartz’s study conducted in the United States of America reported lower sensitivity and specificity for WBC with slightly higher sensitivity and similar specificity for nitrite [16].

 Diabetic patients in our study had slightly higher sensitivity for WBC in comparison to non-diabetic patients, and that is mostly because diabetic patients tend to have recurrent UTI more than non-diabetic patients. However, for nitrite, diabetic patients had lower sensitivity results than non-diabetic patients. Regarding specificity, diabetic patients and non-diabetic patients had similar results for WBC, but nitrite had higher specificity for diabetic than non-diabetic patients.

 To the best of our knowledge, no articles were found that addressed the association of WBC and nitrite sensitivity and specificity with diabetic patients. Indeed, diabetic patients’ results are considered a strength from this perspective. One of the limitations that we found in this research is although dipstick is cost- and time effective, WBC and nitrite in dipstick have varied results among multiple studies in the literature and that makes it difficult to depend on the dipstick urinalysis alone without culture in diagnosing UTIs. More studies are recommended in this field to determine if we can depend on the dipstick urinalysis alone without culture.

## Conclusions

Dipstick urinalysis is a rapid test done to detect UTIs. Our study showed that the results of WBC were more sensitive and specific than nitrite. However, both WBC and nitrite had lower sensitivity and specificity than the gold standard test, which is the urine culture. Diabetic patients had slightly different results than non-diabetic patients. Although dipstick is cost- and time effective, WBC and nitrite in dipstick have varied results among multiple studies in the literature, and that makes it difficult to depend on the dipstick urinalysis alone without culture in diagnosing UTIs. More studies are recommended in this field to determine if we can depend on the dipstick urinalysis alone without culture.
